# Managing a “responsibility vacuum” in AI monitoring and governance in healthcare: a qualitative study

**DOI:** 10.1186/s12913-025-13388-z

**Published:** 2025-09-29

**Authors:** Kellie Owens, Zachary Griffen, Lasya Damaraju

**Affiliations:** 1 Division of Medical Ethics, New York University Grossman School of Medicine, 227 E 30th Street, New York, 10016 NY USA; 2 New York University, New York, NY USA

**Keywords:** Artificial intelligence, Machine learning, Maintenance, Monitoring, Responsibility

## Abstract

**Background:**

Despite the increasing implementation of artificial intelligence (AI) and machine learning (ML) technologies in healthcare, their long-term safety, effectiveness, and equity remain compromised by a lack of sustained oversight. This study explores the phenomenon of a “responsibility vacuum” in AI governance, wherein maintenance and monitoring tasks are poorly defined, inconsistently performed, and undervalued across healthcare systems.

**Methods:**

We conducted semi-structured interviews with 21 experts involved in AI implementation in healthcare, including clinicians, clinical informaticists, computer scientists, and legal/policy professionals. Participants were recruited through purposive and snowball sampling. Interviews were transcribed, coded, and analyzed using abductive qualitative methods to identify themes related to maintenance practices, institutional incentives, and responsibility attribution.

**Results:**

Participants widely recognized that AI models degrade over time due to factors such as data drift, changes in clinical practice, and poor generalizability. However, monitoring practices remain ad hoc and fragmented, with few institutions investing in structured oversight infrastructure. This “responsibility vacuum” is perpetuated by institutional incentives favoring rapid innovation and strategic ignorance of AI failures. Despite these challenges, some participants described grassroots efforts to monitor and maintain AI systems, drawing inspiration from fields such as radiology, laboratory medicine, and transportation safety.

**Conclusions:**

Our findings suggest that institutional and cultural forces in healthcare deprioritize the maintenance of AI tools, creating a governance gap that may lead to patient harm and inequitable outcomes. Addressing this responsibility vacuum will require formalized accountability structures, interdisciplinary collaboration, and policy reforms that center long-term safety and equity. Without such changes, AI/ML technologies designed to improve patient health may introduce new forms of harm, ultimately eroding trust in AI and machine learning for healthcare.

**Supplementary Information:**

The online version contains supplementary material available at 10.1186/s12913-025-13388-z.

## Introduction

With artificial intelligence (AI) and machine learning (ML) billed as the next great innovations in American healthcare, the AI/ML field also faces a widely-recognized “AI chasm”—a significant gap between the development of AI/ML models and their successful integration into clinical practice [1]. There are many reasons why AI systems may fail in practice, including technical failures as models move from controlled environments to assessing live, real-world data. This article instead focuses on some of the social and structural explanations for the AI chasm. Specifically, and in contrast to AI technologists who are primarily attuned to innovation and disruption, our work focuses on the less visible, mundane work of repair and maintenance required for AI technologies to work in practice and be sustained over time.^1^

Based on initial interviews with 21 clinicians, clinical informaticists, computer scientists, and legal/policy experts, we argue that maintenance and repair is often ignored in the AI/ML product pipeline, commonly leading to negligence about the safety and efficacy of AI/ML products over time, as well as the devaluation of invisible labor needed for maintenance and repair. While invisible labor has been theorized by sociologists as care work that is often feminized and located outside formal institutions, alternative rationales are employed in the devaluation of work required to maintain AI/ML infrastructure [2]. Instead, we demonstrate that the emergence of these sociotechnical systems is part of a larger shift [3] away from physicians and toward a range of other professional roles involved in the practice of delivering healthcare and in the building of AI/ML tools. We find that while there is widespread acknowledgement that AI tools can fail, degrade, or produce inequitable outputs over time, there is also a *responsibility vacuum*: multiple professions and sets of expertise are involved in AI implementation, but critical maintenance tasks are unclaimed, undervalued, and inconsistently performed. While ethical deliberation has focused on the importance of keeping a “human in the loop” in AI systems, in healthcare the focus has mostly been on physicians and model developers, despite the workflow of various other actors also being affected [4].

As prior scholarship has argued, this lack of thought and attention to maintenance may be due to Western cultural fascination with newness and innovation [5]. American systems glorify “disrupters” and inventors in funding priorities, professional recognition, and institutional structures that promote breakthrough technologies over the ongoing labor required to sustain them [6, 7]. But focusing so heavily on technology development ignores how these tools get used in reality, as well as how they persist or degrade over time. Understanding persistence and degradation is perhaps particularly critical for ML models, where dataset shift should be expected to cause declines in performance when the data used to train a model begins to look different from the data in a new context [8]. This can include subtle changes in technology (e.g. a new software vendor), changes in population or setting (e.g. a new patient demographic), or changes in behavior (e.g. new reimbursement incentives).

The lack of infrastructure supporting AI model maintenance in healthcare may also be strategic, as institutions are not always held liable for harms they claim to have never known about [9]. If healthcare institutions are not robustly monitoring the performance of AI/ML models over time, they may not be alerted to failures that could lead to actual patient harm but are often difficult to visualize. Because healthcare institutions are often incentivized to remain ignorant about how their tools impact patients [10], building systems to help identify potential harms by characterizing model performance or decline is unlikely to gain robust institutional support. We find that most of our research participants involved with AI/ML development and use in healthcare can describe specific instances of model failures, but that nearly all of these examples involve discovering this decline by chance or in an ad-hoc fashion.

While AI systems are often ignored by developers after implementation, there are also plenty of other actors in AI governance who are creatively (and innovatively!) working to imagine or create new maintenance infrastructure. As Elish and Watkins outline in their notion of repair work, “human labor [is] required to harmonize a technical system with existing organizational and social structures” [11]. In practice, participants in our interview study highlighted the role of clinical staff who recognize when tools work differently than intended and report this to developers. Or, participants would describe how they create new structures of maintenance and repair by drawing on their experience in other domains. Thus, we find that despite a lack of collective attention to processes of maintenance and repair, actors working with AI models on the ground creatively make these tools work in their specific institutional contexts.

In summary, this research characterizes how maintenance and repair of AI systems are enacted. We first argue that maintenance and repair are generally ignored in the responsibility vacuum of medical AI, and we discuss the consequences of that choice. Then, we highlight the invisible labor of healthcare actors to make AI systems work on the ground. We argue that their creativity and innovation in building new infrastructure for maintenance and repair will have great influence on how and whether AI technologies bridge the AI chasm and find persistent use in clinical workflows, especially given the lack of standardized guidelines for the field.

## Materials and methods

The data presented in this manuscript is part of an ongoing exploration of maintenance and monitoring practices for AI/ML tools in healthcare. Data comes primarily from a series of semi-structured interviews with experts who are grappling on a day-to-day basis with how AI/ML models can be both integrated into clinical practice and maintained over time in an ethical and equitable manner. Interview subjects (*n* = 21) include clinical informaticists, clinicians, computer scientists, and legal/policy experts, identified through personal contacts, academic websites, and snowball sampling (demographics provided in Table 1). While all of the experts identified are engaged in academic research in some capacity, many are also involved in the healthcare system as clinicians or legal/regulatory practitioners. Interviews lasted between 30 min and 1 h. Each interview subject completed an informed consent process and was asked about how AI/ML tools factors into their work, what applications of models they consider to be the most promising and/or challenging, and how they view issues surrounding AI/ML including safety, governance, maintenance, and bias (see full interview guide in Supplementary Materials). We stopped conducting interviews when we had reached thematic saturation and no new insights or themes were emerging. The interviews were professionally transcribed and coded by 2 researchers (ZG and LD)() using ATLAS.ti 24 software. Any disagreement between coders was resolved via discussion. Our analysis followed the principles of abductive analysis, a method that builds on grounded theory and emphasizes the importance of generating and refining theories through the systematic examination of empirical data [12]. Additional study details are reported according to the “consolidated criteria for reporting qualitative research (COREQ)” in the Supplementary Materials.

**Table 1:** Participant demographics (*N* = 21) summary of participants self-reported gender identity, race/ethnicity, and job roles

Characteristic		*n*	%
Gender identity			
Men	14		66.7%
Women	6		28.6%
Non-binary	1		4.8%
Race/Ethnicity			
White	10		47.6%
Asian	7		33.3%
Hispanic/Latine	2		9.5%
Black	1		4.8%
More than one race	1		4.8%
Job role			
Clinical informatics	10		47.6%
Computer scientist	3		14.3%
Clinician	3		14.3%
AI Legal/Ethics Expert	5		23.8%
Geographic location			
Midwest	3		14.3%
Northeast	7		33.3%
South	6		28.6%
West	3		14.3%
Non-U.S.	2		9.55%

Percentages are rounded to one decimal place and may not total 100% due to rounding

## Results

In this section we outline the key themes that emerged across our participant interviews. Each theme includes background and context related to how participants discussed this theme, illustrative quotes that represent common participant viewpoints or experiences, and our own analysis of the implications this theme has for AI monitoring and governance.

### AI and problems of drift in healthcare

AI and ML are increasingly being integrated into healthcare systems for a variety of tasks including as aids in diagnosis, clinical decision-making, and promoting operational efficiency. By using datasets like electronic health records and medical imaging databases, AI-powered clinical decision support systems are asked to identify patterns that can assist physicians in diagnosing diseases like cancer, or to optimize hospital workflows by predicting patient deterioration. All of these models are vulnerable to various kinds of “drifting,” which can erode their performance, accuracy, and fairness over time. For example, data drift occurs when there are discrepancies between the data used to train a model and the data it encounters in practice. Shifts in disease prevalence, new treatment guidelines or protocols, and even switching laboratory hardware can cause model predictions to fail. Our interview participants commonly reported this problem:

My model performed really well on [a] certain group. And then when I try it for different sub-groups, then it’s not working… [It is] not just about individuals or health conditions. Even [the type of] scanner seems to have an impact when we try to use the same condition with a different scanner or output. So, we do not even have machine learning models or AI models that are so good that it can identify the same condition regardless of the scanners. - Computer Scientist.

This problem is particularly troubling when models are designed to be used across healthcare systems that look vastly different, such as those developed by electronic health records platforms like Epic:

…We take models, we deploy them across the world. We watch how well they work at [large academic health system]. We have no idea how well they work in the southern Idaho community care center. It’s gonna break in the second place and we’re not gonna notice. And that’s awful. - Legal/Policy Expert.

A large body of research has demonstrated how various types of drift can erode model performance, with a STAT news report suggesting that models like those for sepsis prediction can produce outcomes no better than a coin-flip after just a few years [13]. Sometimes, fixing this problem and improving model performance is fairly trivial, and in other cases models may be wholly broken.

The harms that can arise from data drift are patterned, and generally fall upon groups with less representation in model training datasets, such as those with lower socioeconomic status [14]. Data for healthcare modeling is also heavily skewed by geography. For example, researchers analyzing 5 years of peer-reviewed articles describing AI algorithms for diagnostic tasks in patient care found that 71% used data from California, Massachusetts, or New York. Approximately 60% of the articles only used data from these three states, 34 states were completely unrepresented, and 13 states contributed limited data [15]. Our interview participants also recognized this, as a clinical informaticist noted: “Who are the folks generating stuff in Louisiana, and Arkansas, and Missouri, and Oklahoma? I mean, it’s not really happening.”

Harms arise not just from poor data representation, but also from improper modeling decisions. Most famously, a widely-used algorithm that helps to triage scarce medical resources was found to reduce the number of Black patients who should have been eligible for extra services by more than half when the model used predicted health costs and a proxy for health needs [16].

Thus, while maintenance practices are required for nearly all medical devices and technologies, machine learning and AI models are particularly prone to degradation and bias and will require more extensive efforts to properly maintain. In the following section, we detail existing AI monitoring and maintenance practices, as reported by our research participants.

### AI monitoring and maintenance practices

Mirroring prior literature on maintenance in science and technologies studies, our participants reported that maintenance and monitoring procedures for AI/ML models in healthcare were generally under-emphasized. Most institutions or research groups used ad-hoc methods for ongoing monitoring, although many expressed interest in building a more formal infrastructure. For example, when asked about the post-implementation phase of the AI product lifecycle, a Chief Medical Informatics Officer at a major academic medical center responded:

[Monitoring is] the hardest part…People need to realize that about five years ago, we weren’t monitoring anything. Nobody, like, you weren’t doing anything. So, to an extent, something would be better than nothing. And so early on we said, well, jeez, just check it once a year at least for right now. We need to do something and we’re not doing anything. But now, we need to be more nuanced than that. - Clinical Informaticist.

Or, a physician/scientist at another large academic health system similarly notes:

I think what we do right now is pretty elementary, which is that we put a tool into use. We then look at its performance at some time point down the road and assess whether or not its overall performance metrics match what it originally was published at. So, I would say that is the current state…it’s still not a highly structured thing. - Clinical Informaticist.

This lack of infrastructure means that tools may be failing for extended periods of time without notice. For example, a research participant reported how a broken system in their infrastructure was only identified by chance, after months of failure:

I worked in [medical institution] in the IT department, where the lab system wasn’t working for six months, and nobody noticed. It was generating wrong results for six months. And the only reason we noticed it was [because of] a researcher who was like, ‘This lab data doesn’t make any sense.’ And so, that experience and just knowing that in a lab system, in an EHR system, there’s new configuration all the time. There are people changing things. On a basic level, we will always want to make sure that what we expect to be getting we’re still getting as inputs. - Computer Scientist.

As one of our participants working on AI governance highlighted, this lack of monitoring is not specific to AI/ML—there are many areas in healthcare where additional monitoring infrastructure is viewed as useful by participants: “This is all against a backdrop of the fact that we do a crappy job of ongoing monitoring in health in general.” At the same time, active maintenance of AI tools may be particularly important because their decline can be less visible and may go unnoticed, in comparison to when more obvious defects present in physical medical devices.

While we did not find examples of structured and well-established monitoring or maintenance infrastructure, our participants still discussed the types of monitoring practices they engage in, even if those practices are ad-hoc. Many organizations use model dashboards to track performance metrics, although consensus on what those metrics should be is hard to find [17]. Generally, model dashboards track performance metrics that assess levels of drift, or assess model outputs and performance across relevant subgroups (which usually included performance across race and gender categories):

We do algorithmic drift calculations to see if our predictions are drifting. And they always do. And for various reasons… like there’s seasonal drift. People are a lot more productive on Monday than they are on Friday. There’s even drift within the week, within the month. All that stuff you take into consideration, and you create thresholds. And then when things move out of the thresholds for us, we like to try to retrain models to see if it’s just a matter of either retraining the model and changing the thresholds. Or actually doing more feature engineering and coming up with better models without having to change anything. And that’s all assuming we’re still getting the same inputs. But it’s such a dynamic system because the practice is also changing. Especially the academic medical centers. Practice is changing all the time. Policy is changing. There are quality initiatives going on. There is all this stuff. And so, it’s really a moving target. And so, we just kind of are always looking at that stuff and then trying to decide what we’re gonna do. - Clinical Informaticist.

We encourage manufacturers or AI developers to pre-emptively think about which populations might be disadvantaged or are important to think about…And then, to test for performance within those subgroups. Not just across the aggregate population. And, look for discrepant performance. - Clinical Informaticist.

While describing these ideal procedures for model implementation and monitoring, the same participants also noted: “So, all of that is really like a pie in the sky. Right? That’s what we’d like to do.” We did not find examples of well-resourced and structured maintenance plans for AI in healthcare, even at large academic institutions actively building and implementing this technology, indicating the widespread existence of a responsibility vacuum across the healthcare sector.

### Strategic ignorance and the politics of being “left behind”

We could hypothesize that infrastructure and attention to maintenance of AI models in healthcare is scarce because actors in the relevant fields do not believe there is risk or harm from unmonitored models. Instead, nearly all participants in our study discussed data drift and problems arising from poor data or modeling decisions. Thus, we find that these issues with AI/ML models in healthcare are recognized and well-understood by practitioners in the field. Why, then, did we simultaneously find that there were very few mechanisms to address problems with AI model performance over time? Our research participants frequently discussed this disconnect, suggesting both that there are real and known problems with model performance decline or bias and that many are choosing to de-prioritize or ignore these problems:

The things that we already know about how the modeling works, and the bias that’s encoded in these models, and the bias in the data, and the workflows that are being fed into the models, all of those things. I feel like people know; they really, really know very well that they’re there. You ask anyone long enough and they’ll say, ‘Yeah, I know.’ But then they’re like, ‘I don’t want to be left behind.’ - Clinical Informaticist.

We heard numerous references to the Silicon Valley ethos of “move fast and break things,” and to the general idea that institutions are committed to innovating quickly to avoid being “left behind.” While some participants were optimistic about the fast pace of AI development in healthcare, others highlighted the costs of this choice:

I really feel like when I talk to people [building AI models]…they really think that you can break it first and then fix it. And it really scares me because I think a lot of those people don’t have a lived experience of being disadvantaged. And they don’t understand that what they’re proposing to do is just going to cause a wider gap between those that have and those that have not. So that’s what I really don’t like…I was in a meeting at [institution] where we were arguing…and people said something like…’Why do you care so much about…these models and the biases embedded in them?’ And I was like, ‘I’m Black, my kids are Black, I care. And I’ve seen the data. I see the data. I see when a Black person comes into the hospital less attention is paid to them. That’s what happens. When you’re not a native speaker of English, less attention is paid to you. When you’re a woman, less attention is paid to you.’ I see, it’s in the data, right? But the people who are saying that have never had that lived experience. And so, it doesn’t matter to them. - Clinical Informaticist.

By choosing to develop AI models without mitigating existing and known inequities in health systems data and modeling (and practice), developers are choosing to prioritize speed of product development over equitable impact. Due to their fears of being “left behind” in AI innovation, wealthy institutions building AI models seem to be instead choosing to themselves leave behind the already marginalized. Of course, the quick pace of AI product development could also be met with rapidly developed (and innovative) maintenance infrastructure, but so far this has not been the case.

To understand this trend, we find existing literature on strategic ignorance illuminating. As Linsey McGoey and colleagues have described, strategic ignorance is not merely a lack of knowledge but an active practice—one that is shaped by institutional incentives, professional cultures, and political economies of innovation. McGoey argues that ignorance can be cultivated and maintained when it serves particular interests such as preserving power, avoiding accountability, or sustaining profitable systems [9]. In the case of AI in healthcare, we find that strategic ignorance is visible in the prioritization of rapid innovation while technologists deflect responsibility for the well-documented risks of unmonitored AI models. Our research participants did not dispute the existence of bias, model drift, or the potential harms of poor AI implementation; rather, they framed these issues as secondary concerns compared to the competitive imperative to deploy AI quickly. This suggests that the absence of AI maintenance infrastructure is not simply an oversight, but a structural outcome of innovation-driven institutional priorities that systematically disincentivize ethical concerns in favor of perceived technological progress.

### Visualizing harm in a responsibility vacuum

If institutions are generally incentivized not to build systems that would identify and mitigate harms from implemented AI models, our research then seeks to understand the practices and mechanisms that prevent these harms from being visualized. We find that responsibility for monitoring AI performance is widely diffused, fragmented, and deflected, creating a responsibility vacuum where errors, biases, and harms are difficult to track, attribute, and address. We suggest that this fragmentation is not simply a byproduct of technological complexity but reflects deeper institutional priorities that reinforce innovation over maintenance.

First, we find that health systems are not well-designed to identify harms generally, and perhaps especially for AI models. As a legal scholar in our interview study notes:

Demonstrating causation [for harms] is going to be really challenging…It’s hard to know whether something went right or wrong because of what the physician did or didn’t do as opposed to the fact that you were already sick, which is why you needed healthcare in the first place. When we layer the opacity of algorithmic systems working in the backdrop on top of those, often it’s gonna be problematic…[If an AI model] was wrong for that patient, because it wasn’t adequately trained on their particular ethnic group? That’s gonna be hard to catch. I hope we catch it. Hope we have robust data systems and we recognize these patterns. - Legal/Policy Expert.

As we have argued previously [18], this is particularly true for identifying harms to groups because our systems of research and clinical oversight are almost singularly attuned to monitoring for individual risks and harms. Our research participants suggested that users of AI systems would need to make a concerted effort and go out of their way to identify patterns of poor performance, lest they be nearly impossible to notice:

Maybe it’s just, every now and then nurses who are the users for a hypothetical tool notice that this didn’t work well for a middle age, Muslim women or something like that. But they don’t see that many middle aged Muslim women. And so it’s just like, one nurse notices it here. And then one nurse notices it for this patient. And then a few months later, this patient, and then one other nurse last year had a similar kind of thing. But they might all feel like one offs and how do you have eyes on the whole process to stitch those different things together into a pattern? I think it’s a very tricky problem to solve, which is why I don’t see a lot of people solving it, I think it would be possible with the right level of institutional leadership support, and maybe the right technical monitoring things there.^2^ - Clinical Informaticist

Numerous participants mentioned the importance of technical monitoring tools to help identify performance patterns that humans or end users may not recognize, but few could point to specific products that they were satisfied with in this space.

Relatedly, when we asked participants who should be responsible for ongoing maintenance and monitoring, they were often unsure and would defer to others, citing a lack of expertise or capacity:

For me…the type of researcher that I am, I think it’s not my role to do that [monitoring]. Not to escape accountability for the consequences of the things you develop. But I think that oftentimes the role of the [machine learning] researcher is overloaded with ‘Oh, you need to have a deep understanding of ethics, and a deep understanding of this, and a deep understanding of the deployment…’ But I’m not sure it’s the role of the machine learning researcher to make sure that deployment is going well. - Computer Scientist.

Indeed, machine learning researchers likely do not have the required skills and expertise to monitor clinical deployment outcomes, at least by themselves, further underscoring the existence of a responsibility vacuum in AI maintenance. The participant above went on to describe who *is* responsible for ongoing monitoring, suggesting that while there are many actors involved in AI model development and implementation, none seem well-suited to take on monitoring responsibilities:

The other set of people that I think of is maybe the hospital’s IT team. My impression of hospital IT teams is that they are so weighed down by the legacy software that they have to interact with that they, too, are overwhelmed. And, so, I don’t think that they are the right choice for this job either. So, maybe it does necessitate creating a different role, and obviously a well-compensated role, for maintaining these models. I don’t think that there is an existing actor, like in the whole pipeline, who is a natural fit for it. - Computer Scientist.

Multiple participants mentioned health system IT departments as a potential centralized organization responsible for monitoring or maintaining AI tools, but were skeptical that these departments could succeed without overhaul:

But from my experience, when you’ve got a system, there’s always this tension of who’s going to maintain it. The IT department’s not doing model calibration and building new models. Right? They’re just too busy keeping the lights on. - Computer Scientist.

In addition to IT departments, clinicians are often positioned as the final safeguard in “human-in-the-loop” systems, tasked with monitoring ongoing performance and alerting others to problems. As we have argued in other work [4], clinicians are often ill-suited for this role for a variety of reasons, including a lack of expertise in AI modeling and a lack of capacity to assess performance. Many of the participants in our study agreed:

I’m a little worried that we’re going to rely too uncritically on humans in the loop to solve problems…I think we offload too much of this on to physicians with the idea that, yeah, okay, look, we’re not going to have super robust governance at the local level, like, it’d be nice if we could, but oh, man, that’s resource intense…but don’t worry, at least we have the physician who will check and make sure that it’s, you know, appropriate for their patient and their particular circumstance. And I just am deeply mistrustful…it’s like, you’ve got a soccer team. And you’re like, alright, cool. Everybody off the field except the goalie. The one goalie, way to go, you got this! - Legal/Policy Expert.

Another potential problem our participants identified with relying on a “human in the loop” is how much tacit knowledge gets lost when individuals leave an institution where their work has been vital to system maintenance:

The hardest part that I found…is just keeping the people. In academia, I’m constantly getting new software engineers. New data engineers that are junior because that’s who we can afford. We teach them. Train them up. They get really good. Then they go get a really good job because now they have better skills, and they know stuff. And you can’t slight them for going and getting paid for what they’re worth. Right? - Clinical Informaticist.

High staff turnover represents a significant problem for building successful monitoring procedures, as the people with the greatest knowledge of a model may move quickly onto other projects or to another institution. As this example demonstrates, maintaining a “human-in-the-loop” is not just about system safety but also entails recognizing and rewarding the invisible labor that keeps AI models humming.

As Cambrosio and colleagues theorized, the emergence of regulatory objectivity in biomedicine emphasizes procedural standardization and collective decision-making over individual expertise or scientific certainty [19]. Much of medical practice, such as drug development or clinical trials, relies on guidelines, protocols, and regulatory agencies to establish and manage medical knowledge. For AI development, standardized guidance is still in its infancy, and regulatory agencies like the FDA have struggled to provide oversight of most AI models used by healthcare systems [20]. At the same time, modern biomedicine also introduces ambiguity and inertia as responsibility for decision-making is dispersed throughout a range of actors including regulatory agencies, clinical researchers, industry stakeholders, and healthcare institutions [21]. We find this to be the case in AI implementation and monitoring, where a growing number of professions are involved in a product pipeline that no one takes ownership over. In addition, AI technologies are often intentionally designed to remove decision-making from humans, falsely suggesting that an objective system can make those decisions autonomously. Like simpler, much earlier quantification processes analyzed by Porter [22], AI is thus often implemented to promote “mechanical objectivity” and displace trust in experts. At the same time, when expertise is fractured or devalued, we find that organizational structures obfuscate who should have accountability or responsibility for AI monitoring and maintenance. This culmination of structural and institutional mechanisms, including the perceived benefits of strategic ignorance, leads to a responsibility vacuum. We suggest that it is thus unsurprising that maintenance and monitoring infrastructure are not well-developed, because it is not clear who should be building this infrastructure.

### The invisible creativity of AI maintainers in practice

While monitoring and maintenance practices are not well-developed for AI/ML in healthcare writ large, we nonetheless find many creative examples of individuals and groups working to build new systems to monitor AI in their own institutions. It became clear early in the process of conducting this research that participants had a plethora of ideas about proper AI maintenance and governance. These were introduced largely unprompted, and often through the uses of metaphor and analogical reasoning [23].

For example, participants frequently looked towards the transportation industry for guidance about proper monitoring practices. They drew on metaphors including aviation safety, car service manuals, space travel, and autonomous vehicle navigation systems. This type of analogy was captured most succinctly by a computer scientist:

What we need is essentially a car service manual. Right? Every 5,000 miles you do this. Every 15,000 miles you do this. And it’s very standardized. Right? And, obviously, not everyone follows that, but it gives you kind of a service manual for exactly what the intervals are and, so that’s probably gonna be in the future. Every three months we examine data drift and we examine the performance of this model. - Clinical Informaticist.

Other participants referenced adjacent medical fields and oversight mechanisms when envisioning new AI maintenance systems:

I think there are parallels to when the X-ray was first invented. X‑rays were a new and exciting technology, but then the entire clinical field of radiology emerged around that technology to ensure that we were using X-rays and CAT scans and MRIs informed by an understanding of disease and patient experience and held to the same clinical standards that we would other kinds of clinical interventions to develop an expertise and a field that balances knowledge about the underlying methods and technologies, but also still is deeply rooted in clinical medicine and patient care. - Clinician.

While the FDA is the primary regulator of AI models when they are considered as medical devices, the agency has limited jurisdiction over much of the AI landscape. This has resulted in a number of participants envisioning how other agencies could get involved in AI regulation, and how those agencies could work with local health systems^3^:

So far, CMS [Centers for Medicare and Medicaid Services] has not stepped into the ring when it comes to regulation of AI devices…but CMS actually is highly involved in the regulation of laboratories, hospital laboratories around the country through CLIA [Clinical Laboratory Improvement Amendments]…And what it means is that local laboratories are responsible for locally…validating and overseeing FDA-approved laboratory tests, and there’s a recognition that just because a test is validated and developed across the country in Stanford, it still needs close attention when we deploy it here [at academic medical center]…As part of the CLIA certification, hospitals get inspected. They’re also required to appoint a CLIA director, so there’s a single point of contact at each hospital for a CLIA certified laboratory, and that person can certainly delegate some of those responsibilities, but there are certain skill sets and reports they have to file to really maintain and document the oversight, testing, evaluation of these different laboratory tests. So, I think that kind of model really is very analogous to thinking about AI/ML systems. And [colleagues and I] were working on [promoting that model]. - Clinical Informaticist.

This thinking represents the less visible “repair work” required to implement and maintain AI technologies designed to “disrupt” clinical workflows [11]. This work includes attempts to establish technical tooling and metrics to assess model performance over time, but has also focused on the people and processes needed to ethically and effectively implement new models. While often unsure how this governance should proceed, respondents noted the importance of gathering the right people “in the room:”

I wish anyone had good guidance…I think you just sort of go with your gut and assemble the right multi-stakeholder kind of team such that if you do realize you got it wrong, then you can all share the blame together. And, you can say, ‘I was including the patient voices. I was including the nurses’ voices’. And, in retrospect, we should’ve done this. But, this was our best guess at the time. This wasn’t just me being an executive decision maker. - Computer Scientist.

These examples illustrate that, despite the lack of formalized AI maintenance structures, there is significant grassroots innovation happening within healthcare institutions as practitioners, researchers, and administrators work to fill gaps in AI oversight. Comparisons to transportation, radiology, and laboratory regulation reflect an implicit recognition that AI governance requires structured, ongoing accountability, rather than the one-time validation processes that currently dominate regulatory approaches. Yet these efforts remain largely informal, underfunded, and institutionally precarious, often reliant on the initiative of individuals rather than being embedded within healthcare systems.

## Discussion

Our findings suggest that while AI/ML-based healthcare technologies are widely acknowledged to degrade over time, the structures necessary to maintain and monitor these tools remain underdeveloped. This absence of formal maintenance infrastructure is not merely a technical oversight but a social and institutional choice, shaped by cultural, economic, and regulatory forces that prioritize innovation over long-term stewardship. The result is a responsibility vacuum, where no single actor or institution claims ownership over AI maintenance, leaving critical tasks—such as bias mitigation, performance recalibration, and failure detection—to be addressed in an ad hoc manner or not at all. Strategic ignorance plays a central role in this dynamic, as institutions benefit from not knowing when AI systems fail, thereby shielding themselves from liability and regulatory scrutiny.

Yet even in the absence of formalized AI governance, we find significant creativity among practitioners who recognize the need for sustained AI oversight. Clinicians, informaticists, and policymakers are drawing on models from transportation safety, radiology, and laboratory oversight, among many others, to envision new approaches to AI maintenance. Their work highlights the necessity of institutionalizing repair work, rather than treating AI failures as isolated or exceptional events.

The ethical implications of our findings are substantial. AI systems in healthcare are often presented as objective and scalable solutions to long-standing problems, but our data highlight how their real-world use is deeply shaped by institutional and professional dynamics that can lead to safety concerns or reinforce inequities. When maintenance infrastructure is lacking and responsibilities are diffuse, the harms of degraded model performance—such as biased recommendations, diagnostic errors, or inappropriate resource allocation—often fall disproportionately on historically marginalized populations. Moving forward, regulatory bodies, healthcare institutions, and professional organizations should prioritize AI maintenance infrastructure as a fundamental component of patient safety and clinical efficacy. Without intentional investment in this infrastructure, the technologies designed to improve patient health will introduce new forms of harm, ultimately eroding trust in the promise of AI and machine learning for healthcare.

This study has several limitations. First, while we sought to include diverse roles and institutions in our sample, this is a small, qualitative interview study with limited size and scope. While qualitative interviews provide rich, in-depth insights into participants’ perspectives, we cannot be certain that the perspectives reflected in our data are generalizable outside of our sample. That said, we stopped conducting interviews when no new themes or insights were emerging and we had reached saturation. Second, the study relies on self-reported experiences, which may be shaped by participants’ positions within their organizations and their willingness to speak openly about failures or institutional shortcomings.

Building on this work, future research should explore the design and evaluation of formalized AI maintenance structures. Interdisciplinary collaborations between technologists, ethicists, clinicians, and policymakers could help prototype new roles, workflows, and governance models. Additionally, examining how different regulatory approaches—such as those in the EU, U.K., Canada, or Australia—handle ongoing AI oversight could offer valuable insights for reforming AI monitoring practices. Finally, there is a need for longitudinal studies that follow AI systems after deployment to trace patterns of failure, repair, and institutional response over time.

## Conclusion

In this study, we analyse the views of numerous experts whose work is affected by the absence of AI maintenance infrastructure in healthcare. In addition to observing the existence of a responsibility vacuum, our findings suggest that creative solutions to the lack of monitoring practices often emerge when different stakeholders draw from neighbouring fields and systems. As new use cases for AI models are identified and scaled up in healthcare, this study suggests that even without more official standardized guidance, developing responsible maintenance and monitoring practices can still occur through more local, collaborative efforts.

## Supplementary Information

Supplementary Material 1.

Supplementary Material 2.

## Data Availability

Due to the identifiable nature of the data used in this study, it will not be made publicly available. Metadata such as the interview guide is available in Supplementary Materials.

## Footnotes

1 We can distinguish between three different phases of the AI model lifecycle: (1) development, which includes planning, design, data collection and processing, building, and testing the model, (2) deployment, which is the process of introducing models into clinical settings, and may include additional validation, and (3) maintenance and monitoring, which occurs after models are operational. Our focus in this study is on the third phase.

2 AI developers in healthcare generally agree that it is important to test and monitor model performance across demographic groups to assess differences. While the underlying motivation for this testing seems to relate to concerns of equity and fairness, we also heard numerous examples where participants assume that they will find differences between social groups due to inherent biological differences between them. For example, a respondent said: “So, the bony anatomy among different ethnicity, among different races, could be also different. If you look at the face of a Chinese guy, and look at the face of a Persian guy like me, and look at the face of a Japanese guy, and Vietnam guy, an American guy or a United Kingdom kind of lady or whatever: so, our face, the structure of the face is different, pretty much different. If we want to make an AI model to segment those kinds of bony anatomies objectively, automatically, we also need to make those components fair, to address all people in the same way.” A focus on inclusion and diversity in clinical medicine can end up reifying biological differences between social groups [24] , and we see clear evidence of this from AI developers and users.

3 Federal regulation of AI and medical devices has weakened significantly since the beginning of the Trump administration. While participants envisioned other federal regulatory systems getting involved in AI governance, changes in administrative priorities makes this less likely.

## References

[1] Aristidou A, Jena R, Topol EJ. Bridging the chasm between AI and clinical implementation. The Lancet. 2022;399:620. 10.1016/S0140-6736(22)00235-5 35151388

[2] Daniels AK. Invisible work. Soc Prob. 1987;34:403–15.

[3] Epstein S, Timmermans S. From Medicine to Health: The proliferation and diversification of cultural authority. J Health Soc Behav. 2021;62:240–54. 34528483 10.1177/00221465211010468

[4] Griffen Z, Owens K. From, “Human in the Loop” to a Participatory system of governance for AI in healthcare. The Amer J Bioeth. 2024;24:81–3. 39226015 10.1080/15265161.2024.2377114

[5] Russell AL, Vinsel L. After innovation, turn to maintenance. Technol Cult. 2018;59:1–25. 29731465 10.1353/tech.2018.0004

[6] Schaetz N, and Schjøtt A. AI Hype and its Function: An Ethnographic Study of the Local News AI Initiative of the Associated Press. Digital Journalism. Published online January 29, 2025.

[7] Sheehan P. To the Moon: Hype and Start-Up Work. Contexts. 2024;23:26–31.

[8] Finlayson SG, Subbaswamy A, Singh K, Bowers J, Kupke A, Zittrain J, et al. The clinician and dataset shift in artificial intelligence. New England J Med. 2021;385:283–6. 34260843 10.1056/NEJMc2104626 PMC8665481

[9] McGoey L. The logic of strategic ignorance. The British J Soc. 2012;63:533–76. 22950467 10.1111/j.1468-4446.2012.01424.x

[10] Heimer CA. Inert facts and the illusion of knowledge: strategic uses of ignorance in HIV clinics. Econ Soc. 2012;41:17–41.

[11] Elish MC, Watkins EA. Repairing Innovation: A study of integrating AI in clinical care. Data & Soc. https://datasociety.net/library/repairing-innovation/. Published September 30, 2020. Accessed 10 Apr 2025.

[12] Timmermans S, Tavory I. Theory Construction in Qualitative Research: From grounded theory to abductive analysis. Soc Thry. 2012;30:167–86.

[13] Ross C. AI gone astray: How subtle shifts in patient data send popular algorithms reeling, undermining patient safety. STAT. 2022. https://www.statnews.com/2022/02/28/sepsis-hospital-algorithms-data-shift/. Accessed 10 Apr 2025.

[14] Gianfrancesco MA, Tamang S, Yazdany J, Schmajuk G. Potential Biases in Machine Learning Algorithms Using Electronic Health Record Data. JAMA Intern Med. 2018;178:1544–7. 30128552 10.1001/jamainternmed.2018.3763 PMC6347576

[15] Kaushal A, Altman R, Langlotz C. Geographic distribution of us cohorts used to train deep learning algorithms. JAMA. 2020;324:1212–3. 32960230 10.1001/jama.2020.12067 PMC7509620

[16] Obermeyer Z, Powers B, Vogeli C, Mullainathan S. Dissecting racial bias in an algorithm used to manage the health of populations. S. 2019;366:447–53. 31649194 10.1126/science.aax2342

[17] Feng J, Xia F, Singh K, Pirracchio R. Not all clinical ai monitoring systems are created equal: Rev Reco. NEJM AI. 2025;2(2).

[18] Chapman CR, Quinn GP, Natri HM, Berrios C, Dwyer P, Owens K, et al. Consideration and Disclosure of Group Risks in Genomics and Other Data-Centric Research: Does the Common Rule Need Revision? Am J Bioeth. 2025;25:47–60. 38010648 10.1080/15265161.2023.2276161 PMC11167719

[19] Cambrosio A, Keating P, Schlich T, Weisz G. Regulatory objectivity and the generation and management of evidence in medicine. Soc Scie Med. 2006;63:189–99. 10.1016/j.socscimed.2005.12.007 16455171

[20] Lawrence L. The FDA plans to regulate far more AI tools as devices. The industry won’t go down without a fight. STAT. 2023. https://www.statnews.com/2023/02/23/fda-artificial-intelligence-medical-devices/. Accessed 10 Apr 2025.

[21] Clarke AE, Shim JK, Mamo L, Fosket JR, Fishman JR. Biomedicalization: technoscientific transformations of health, illness, and U.S. biomedicine. Amer Soc Rev. 2003;68:161–94.

[22] Porter TM. Trust in numbers: the pursuit of objectivity in science and public life. Princeton, N.J.: Princeton University Press; 1995. 10.1177/030631299029004007 11623934

[23] Vaughan D. Analogy, Cases, and Comparative Social Organization. In: R Swedberg, ed. Theorizing in Social Science: The Context of Discovery. Redwood City, CA: Stanford University Press; 2014;61–84.

[24] Epstein, S. Inclusion: The Politics of Difference in Medical Research. Chicago, IL: University of Chicago Press; 2007.

