# Supplementary material for: Managing a “responsibility vacuum” in AI monitoring and governance in healthcare: a qualitative study
Source: BMC Health Serv Res. 2025 Sep 29;25:1217. doi: 10.1186/s12913-025-13388-z (PMC12482494; doi:10.1186/s12913-025-13388-z)
Supplement: Supplementary file 1 — Supplementary Material 1. [file 12913_2025_13388_MOESM1_ESM.docx]

**Supplemental Materials S1**

**Consolidated criteria for reporting qualitative studies (COREQ): 32-item checklist**

Derived from: Tong, A.; Sainsbury, P.; Craig, J. Consolidated criteria for reporting qualitative research (COREQ): a 32-item checklist for interviews and focus groups. *Int J Qual Health Care* **2007**, *19*, 349-357, doi:10.1093/intqhc/mzm042.

| **No. Item** | **Guide questions/description** | **This Research** |
| --- | --- | --- |
| **Domain 1: Research team and reﬂexivity** | | |
| *Personal Characteristics* | | |
| 1. Interviewer/ facilitator | Which author/s conducted the interview or focus group? | *Owens (lead), Griffen, Damaraju* |
| 2. Credentials | What were the researcher’s credentials? E.g. PhD, MD | *PhD, MSME (Owens), PhD, MA (Griffen), BS in progress (Damaraju).* |
| 3. Occupation | What was their occupation at the time of the study? | *Assistant Professor of Medical Ethics (Owens), Postdoctoral Fellow in Medical Ethics (Griffen), Undergraduate at New York University (Damaraju)* |
| 4. Gender | Was the researcher male or female? | *The researchers were female (Owens & Damaraju) and male (Griffen).* |
| 5. Experience and training | What experience or training did the researcher have? | *Dr. Owens is a sociologist and medical ethicist with expertise in qualitative research and in-depth interviewing. Dr. Griffen is a sociologist with expertise in qualitative research and analysis. Ms. Damaraju was an undergraduate student building training in qualitative methods and empirical bioethics.* |
| *Relationship with participants* | | |
| 6. Relationship established | Was a relationship established prior to study commencement? | *No prior relationship.* |
| 7. Participant knowledge of the interviewer | What did the participants know about the researcher? e.g. personal goals, reasons for doing the research | *Professional background, research interests, reasons for doing this research.* |
| 8. Interviewer characteristics | What characteristics were reported about the interviewer/facilitator? e.g. Bias, assumptions, reasons and interests in the research topic | *Dr. Owens was the primary interviewer and led all interviews. Her training as a sociologist lends her a critical analytical toolkit, and her identity as a white, cis-het woman likely influences participant interactions.* |
| **Domain 2: study design** |  |  |
| *Theoretical framework* | | |
| 9. Methodological orientation and Theory | What methodological orientation was stated to underpin the study? e.g. grounded theory, discourse analysis, ethnography, phenomenology, content analysis | *This study uses the principles of abductive analysis. Abductive analysis builds on grounded theory and is popular in medical sociology and other social sciences. It is a method of inquiry that emphasizes the importance of generating and refining theories through the systematic examination of empirical data. The process is iterative, encouraging researchers to be flexible in their thinking, to remain continually engaged with their data, and to be prepared to adjust their theoretical insights in light of new evidence. This approach is fundamentally different from deductive reasoning, which starts with a theory and then seeks evidence to confirm or refute it, and inductive reasoning, which starts with observations and then generates a theory. Like grounded theory, abductive analysis provides a framework for sampling, coding, and analysis, and directs researchers to iteratively analyze data throughout the data collection and analysis process.* |
| *Participant selection* | | |
| 10. Sampling | How were participants selected? e.g. purposive, convenience, consecutive, snowball | *Purposive and snowball sampling* |
| 11. Method of approach | How were participants approached? e.g. face-to-face, telephone, mail, email | *Contacted by e-mail by Dr. Owens* |
| 12. Sample size | How many participants were in the study? | *21* |
| 13. Non-participation | How many people refused to participate or dropped out? Reasons? | *46 people did not respond to email requests or declined an interview. No one dropped out after beginning participation.* |
| *Setting* | | |
| 14. Setting of data collection | Where was the data collected? e.g. home, clinic, workplace | *At participants’ homes or in their offices, online via Zoom.* |
| 15. Presence of non-participants | Was anyone else present besides the participants and researchers? | *No* |
| 16. Description of sample | What are the important characteristics of the sample? e.g. demographic data, date | *10 participants were clinical informaticists, 3 participants were computer scientists, 3 participants were clinicians, and 5 participants were legal/policy experts. Participant demographics: Gender: 14 Men, 6 Women, 1 Non-Binary. Race/Ethnicity: 10 White, 7 Asian, 2 Hispanic/Latine, 1 Black, 1 More than One Race. Interviews were conducted between 2023-2024.* |
| *Data collection* | | |
| 17. Interview guide | Were questions, prompts, guides provided by the authors? Was it pilot tested? | *Dr. Owens developed the interview guide, provided in the supplemental material S2. The interview guide was pilot tested internally with Dr. Owens’ colleagues, and revised accordingly.* |
| 18. Repeat interviews | Were repeat interviews carried out? If yes, how many? | *No* |
| 19. Audio/visual recording | Did the research use audio or visual recording to collect the data? | *Audio and video recording* |
| 20. Field notes | Were ﬁeld notes made during and/or after the interview or focus group? | *Yes* |
| 21. Duration | What was the duration of the inter views or focus group? | *Approximately 30 minutes to 1 hour.* |
| 22. Data saturation | Was data saturation discussed? | *Yes, interviews stopped once data saturation was on the topic of maintenance practices.* |
| 23. Transcripts returned | Were transcripts returned to participants for comment and/or correction? | *No* |
| **Domain 3: analysis and ﬁndings** | | |
| *Data analysis* | | |
| 24. Number of data coders | How many data coders coded the data? | *Two (Griffen and Damaraju)* |
| 25. Description of the coding tree | Did authors provide a description of the coding tree? | *No* |
| 26. Derivation of themes | Were themes identiﬁed in advance or derived from the data? | *Both* |
| 27. Software | What software, if applicable, was used to manage the data? | *Atlas.ti* |
| 28. Participant checking | Did participants provide feedback on the ﬁndings? | *No* |
| *Reporting* | | |
| 29. Quotations presented | Were participant quotations presented to illustrate the themes/ﬁndings? Was each quotation identiﬁed? e.g. participant number | *Participant quotations are included and identified only by professional training, to preserve anonymity.* |
| 30. Data and ﬁndings consistent | Was there consistency between the data presented and the ﬁndings? | *Yes* |
| 31. Clarity of major themes | Were major themes clearly presented in the ﬁndings? | *Yes* |
| 32. Clarity of minor themes | Is there a description of diverse cases or discussion of minor themes? | *Yes* |
