# Supplementary material for: Managing a “responsibility vacuum” in AI monitoring and governance in healthcare: a qualitative study
Source: BMC Health Serv Res. 2025 Sep 29;25:1217. doi: 10.1186/s12913-025-13388-z (PMC12482494; doi:10.1186/s12913-025-13388-z)
Supplement: Supplementary file 2 — Supplementary Material 2. [file 12913_2025_13388_MOESM2_ESM.docx]

**PRINCIPAL INVESTIGATOR:** Kellie Owens, PhD

**PROTOCOL TITLE:** Building Equitable Systems of Maintenance and Repair for AI in Healthcare

This guide provides a list of questions that will be asked during the semi-structured interviews. The list is not exhaustive, and the questions will be tailored to fit the conversation. Any changes to the questions and data collected, will be submitted to the IRB for review and approval prior to use.

*Introduction:*

- How would you describe your (medical/scientific/work) specialty and interests?
- What does a typical work week look like for you?
- How do you engage with artificial intelligence or machine learning in healthcare?
- What kind of technical training have you received regarding AI/ML?
  - What do you wish you knew more about in regards to AI/ML?

*Attitudes Regarding Artificial Intelligence and Machine Learning in Healthcare:*

- What excites you about using artificial intelligence and machine learning in healthcare?
- What makes you nervous about artificial intelligence and machine learning in healthcare?
- Please share a few examples of when AI/ML has been useful for [you/a patient/a clinical scenario].
- Please share a few examples of when AI/ML has NOT been useful for [you/a patient/a clinical scenario].
- How do you (or your team/institution) assess, maintain, and repair AI/ML models over time?
- What infrastructure do you think is missing that would improve the way AI/ML models operate over their entire lifecycle?
- Do you have any metrics or frameworks to assess when an AI/ML model is no longer functioning properly?
- Who in your institution is responsible for maintaining the proper function of AI/ML tools over time?
- How do you think AI/ML could affect equity in healthcare outcomes?
- What are the best ways to assess and address any potential inequities caused by AI/ML models over time?
- How do you conceptualize what makes an AI/ML model fair?
- What could be done to improve responsibility and accountability for ensuring AI/ML models produce fair outcomes?

*Demographics*

- How would you categorize your gender?
- How would you categorize your race and ethnicity?
- What is your age?

*Closing:*

- We are studying how stakeholders understand the benefits and risks of AI/ML-based clinical decision support tools, and how to best maintain those tools over time. What else do you think I should have asked you about this topic?
- Is there anything else you would like to add?
